# Mosquito pollination of plants: an overview of their role and an assessment of the possible contribution of disease vectors

**DOI:** 10.1007/s11248-024-00394-w

**Published:** 2024-08-22

**Authors:** Woodbridge A. Foster

**Affiliations:** Department of Evolution, Ecology and Organismal Biology and Department of Entomology, The Ohio State University, Columbus, OH 43210 USA

**Keywords:** Plants, Mosquitoes, Flowers, Pollination, Nectar, Disease vectors

## Abstract

Mosquitoes visit flowers to obtain sugar or other nutrients and therefore possibly serve as major or minor pollinators of some plant species. They also often derive plant nutrients from other sources, such as extrafloral nectaries and honeydew. In a few cases, the plant-mosquito relationship is close, and mosquito pollination has been confirmed. Most plant species visited by mosquitoes, however, appear to depend on multiple means of pollination, particularly other flower-feeding insects. In addition, most mosquito species visit the flowers of many kinds of plants, possibly dispersing pollen in both biologically meaningful and irrelevant ways. This apparent lack of selectivity by both plants and mosquitoes liberates each of them from dependence on an unreliable pollen vehicle or nutrient source. A hypothetical pollinating role for the two top vectors of devastating human-disease pathogens, *Anopheles gambiae* or *Aedes aegypti*, relies on indirect evidence. So far, this evidence suggests that their participation in pollen transfer of native, introduced, or beneficial plants is negligible. The few plant species likely to be pollinated by these vectors are mostly invasive, harmful weeds associated with humans. That conclusion draws support from four characteristics of these vectors: (1) the numerous alternative potential pollinators of the flowers they visit; (2) their common use of diverse *non*-floral sources of nutrients; (3) the females’ infrequent sugar feeding and heavy reliance on human blood for energy; and (4) their relatively low population densities. From these traits it follows that focused suppression or elimination of these two vectors, by whatever means, is highly unlikely to have adverse effects on pollination in endemic biotic communities or on ornamental plants or food crops.

## Introduction

Globally, there are approximately 3500 described species of mosquitoes. Do any of them play pivotal or contributing roles in food chains and food webs, and if so, in which ways? Are mosquitoes important components of biotic communities and ecosystems, either by providing prey for consumers or by keeping their own food sources under control? Or do they at least assist in this regard? These are reasonable balance-of-nature questions. In practical terms, such as pest control or public health, one may ask whether communities of plants and animals will be altered if a particular disease-vector species is greatly reduced or eliminated. That could happen if other organisms are deprived of a necessary food supply or are no longer kept in check by a mosquito’s blood-feeding and pathogen-transmission activities. These bottom-up and top-down mechanisms that determine population density typically are thought of in terms of predation and parasitism. Overlooked are the possible mutualistic relationships between mosquitoes and various organisms with which they interact. Because adults often visit flowers, one such possible plant-mosquito mutualism is pollination of plants. Are mosquitoes important to some native plant communities, to introduced plant species, or to crops and ornamental plants? In addressing that question, this paper will examine mosquito pollination in general, then will focus on two species that are particularly devastating to human health and often are targeted for suppression or are even considered for elimination: *Anopheles gambiae* and *Aedes aegypti*.

First, this assessment presents terminology and descriptions of plant pollination and pollinators, to address the possible role of mosquitoes, because the criteria used to define a pollinator are essential to the issue. Second, it evaluates the role that mosquitoes may play in pollination, with an emphasis on two top vectors of human disease. This topic has become a serious ecological and public health issue, a response to recent advances in genetic methods of mosquito control. These methods have the potential to suppress or even remove a single vector species from a defined geographical area. The following assessment of mosquito-plant relationships and their consequences, presented here, is a synthesis of selected published literature, the thoughts and observations of colleagues, and the provisional conclusions of the author after 50 years of field and laboratory research on mosquito biology and behavior.

The following five questions must be addressed: (1) Flower Visitors: Which mosquito species visit flowers, and which of them are possible, probable, or confirmed pollinators? (2) Circumstantial Evidence: Which observations support the hypothesis that certain mosquito species are likely pollinators? (3) Experimental Evidence: Which mosquito species have been used in controlled experiments under natural conditions to test the hypothesis that a mosquito species is a pollinator? Is the evidence sufficiently strong or convincing to accept this hypothesis? (4) Application of Evidence: Do the above lines of evidence apply specifically to the two top vectors of human disease? Is the evidence for their pollinating abilities sufficiently strong and convincing to justify restraining vector-control methods? (5) Elimination Outcomes: Are the plant species that are sometimes or often pollinated by disease vectors essential to maintain the diversity and stability of the plant communities they occupy? Would suppression or elimination of one of those vectors weaken the population of its plant hosts or upset those communities?

### A. Basics of mosquito pollination potential

Mosquitoes (Diptera: Culicidae) of both sexes ingest sugar and other nutrients from a variety of sources: flower nectar, extrafloral nectaries, hemipteran honeydew, overripe fruit, damaged plant tissue and seed pods, leaf guttation, and even vascular sap from undamaged plant parts in some cases (Foster 1995, 2022). Because they sometimes contact a flower’s anthers and stigmas when extracting its nectar, they may incidentally acquire either pollen grains or pollinia (pollen packets) on their bodies. By this means they could transfer pollen between anthers and stigmas on the same flower (autogamy or self-pollination), on different flowers of the same plant (geitonogamy), or among different plants of the same species that they visit (heterogamy or cross-pollination). Plants that require this transfer, by whatever mechanism, will not set seed or develop fruit without it. Though some species are wind cross-pollinated, by far the most common cross-pollinators are insects, so all species of insects that visit flowers, of which there are many thousands—either for their nectar, for excess pollen or other useful substances, or by deceptive mimicry to resemble mates or carrion—are possible pollinators. Anthophilous (flower loving) mosquitoes conceivably are among them. However, despite the many observations of flower-mosquito associations in some parts of the world, pollination by any mosquito species has been speculated or inferred only rarely, and even more rarely supported by evidence or demonstrated experimentally. Usually, the published observations that might be used to implicate any mosquito species in pollen transfer, let alone a disease vector, are circumstantial. More often, the possibility of pollination is never addressed.

### B. Criteria of pollinators

To demonstrate conclusively that an anthophilous mosquito species plays a significant role in pollinating a particular plant species in nature, we need to show a decline in seed set or other measure of plant reproductive success when a mosquito population is absent or greatly reduced in numbers. In such an experiment, all other variables must remain the same, including the other potential pollinators. The contribution of the domesticated honey bee is an example (e.g., Fajardo et al. 2008). For mosquitoes, this high standard has yet to be met, though there have been opportunities, such as when mosquitoes invade new regions or are removed from them. Experiments such as the bagging of flowers in the field, to exclude mosquitoes, or alternatively to hold the flowers only with mosquitoes, are good indicators of possible natural pollination (see below). They are compromised by the exclusion of all other natural visitors of the same flowers and thus cannot establish the relative importance of a potential pollinator. This is a critical omission.

1. *Classification.* In an attempt to set out specific criteria as evidence for pollination, Adams and Lawson (1993) placed insect-orchid records in three categories: (i) *Suggested*: the flower is visited. (ii) *Probable*: pollen is picked up. (iii) *Confirmed*: pollen is picked up and deposited on the flower of another plant of the same species—to which may be added that fertilization and seed-set are achieved. These are useful criteria, though they require careful observation in the field. For mosquitoes, little has been done to confirm pollination even by these general criteria, so we are left with a set of pollination-promoting characteristics to assess a plant-mosquito relationship under natural conditions, and therefore to gauge the *likelihood* that one or more mosquito species contributes to the reproductive success of a particular plant species in the field. Thus, it is an informed inference. Below are guidelines that assist in that assessment.
2. *Attributes.* Important pollinators should have at least a few of the following attributes: (i) They visit flowers of some species frequently, contact the anthers and stigmas within the flower, and pick up and deposit pollen while doing so. (ii) They move frequently among different individual plants during a bout of foraging, to facilitate cross-pollination. (iii) They tend toward plant specialization (termed monolectic or oligolectic), inclined to visit flowers of one or very few plant species, at least in one foraging bout, i.e., flower constancy. Constancy confers efficiency, so that pollen is not widely distributed among other plant species where it would have no function. (iv) If they are generalists (polylectic) and visit generalist flowers, they may nonetheless be important to some plants, especially if they occur in very large numbers or if their seasonality corresponds to blooming periods of particular plant species, enhancing the probability that they will transfer some pollen to conspecific flowers. (v) They are among only a few flower visitors of that plant species in an area, such that they contribute disproportionately to pollination, rather than coexisting with a wide variety of other visitors that are themselves likely to be promiscuous flower visitors.
3. *Finer Rankings.* When evaluating probable or confirmed pollinators and their attributes listed above, finer conceptual rankings than the three presented by Adams and Lawson (1993) (above) can be useful. Below are two separate scales on which a flower visitor may be ranked along continua between extremes of both *efficiency* and *effectiveness*. The descriptors that can be assigned to hypothetical points on each continuum are these: *important, minor, trivial, redundant,* and *nil*. *(i) Efficiency:* A *highly efficient* pollinator, typically but not always a specialist, accomplishes seed set to the greatest extent possible, by transferring pollen between flowers of only one plant species. A pollinator with *low efficiency* picks up and transfers pollen within one plant species, but only in small amounts or occasionally, resulting in low seed set. The efficiency of pollen transfer by mosquitoes apparently has not been measured, with the exception of specialist orchid-*Aedes* relationships (see below*). (ii) Effectiveness:* An *effective* pollinator, on the other hand, enhances seed set by some measurable amount, large or small, that affects the plant’s reproductive success. An accurate usage of terminology is presented and evaluated by Inouye et al. (1994). It requires an intimate knowledge of mosquito-plant pollen transfer, such as pollen load and loss and the frequency of flower visits to different plants. This information is far beyond the level of detail currently available for mosquitoes.

### C. Mosquito-flower relationship variations

1. *Specialists* They tend to be both highly efficient and highly effective. Their mouthparts or sensory abilities allow them to find and enter the flower or to feed on the floral nectar of only one or a few plant species to which those specialists are adapted through evolved or co-evolved tactics. Such close relationships are rare among mosquitoes, whose relatively short proboscises cannot access the nectar in long corollas or other specially configured flower structures. Narrowly focused flower odors (see below) and the daily timing of nectar flow also can serve as exclusionary factors for mosquitoes. The best documented of these specialized relationships involve specific floral odors and structures that facilitate attraction and pollination by a narrow assemblage of monolectic or oligolectic flower visitors. The best studied examples of specialist associations involve orchids (*Platanthera* spp*.*) of northern North America, which are visited by a few mosquito species in the genus *Aedes*. Other *Platanthera* species also show indications of pollination by mosquitoes (see Peach and Gries 2020). Spanish catchfly (*Silene otites*) of northern Europe also may have a fairly close relationship with a small group of mosquitoes and moths in at least one location in some seasons, but many other insects also visit its flowers, including unstudied diurnal visitors. Likewise, the orchid-mosquito pollination relationship in the genus *Pterostylis* in Australia and New Zealand is narrow, though fungus gnats appear to be the primary pollinators (see more, below).
2. *Flower Constancy*. Fidelity is sometimes behavioral. For example, honey bees, which are well established pollinators, tend to concentrate on one plant species during each foraging trip. Learning (i.e., positive associative conditioning) can play a role in narrowing the responsiveness of mosquitoes to flower cues associated with a nectar reward. Constancy also may be incidental when, for example, large numbers of plants are in bloom at the same time and place as abundant mosquitoes. Associations between particular species of plants and the mosquitoes that visit their flowers have been reviewed by Peach and Gries (2016).
3. *Generalists* At the other extreme are the polylectic (promiscuous) flower-visiting insects that obtain nectar from plants with unspecialized flowers. Generalist plants normally produce large, visually apparent or fragrant clusters of flowers or florets that have short or very short corollas and yield only small amounts of nectar, inducing the insects to move frequently over the flower cluster and between plants. Stable generalist plants can be important to plant-pollinator networks in biotic communities (see Zografou et al. 2020), but the contribution of each type of insect may be either substantial or quite small. Such plants typically achieve effective pollination only by inviting large numbers of a great variety of insects, including flies, bees, wasps, butterflies, moths, and beetles, and in some cases birds, bats, and marsupial mammals. At the same time, the mosquitoes will be visiting a wide variety of plant species. In such cases, mosquitoes are likely to be low on the effective scale, perhaps playing only a small role, if not a redundant one. Most of the pollen that these diverse visitors might pick up and then transfer to other flowers of various plant species is wasted unless one plant species is aggregated in space and time, yet genetically diverse, to promote cross fertilization. And even then, due to plant competition for pollinators, flower aggregation often appears to reduce pollination efficiency of some visitors (Barley et al. 2022).
4. *Flower Visitation vs. Pollination* Attraction to a flower, by itself, is not a reliable indicator of a pollinator. Some flowers are visually or olfactorily attractive to mosquitoes, but the mosquitoes fail to serve as pollinators. A clear example is the common North American and European pest mosquito *Ae. vexans,* which is strongly attracted to the fragrance of common milkweed, *Asclepias syriaca*, in eastern North America and readily feeds on the nectar of its pale pink florets (Sandholm and Price 1962; Yee et al. 1992; Otienoburu et al. 2012). But this mosquito is incapable of detaching the pollinia and therefore cannot deliver them to another flower. That function is performed by heavy-bodied insects such as bumble bees, honey bees, wasps, and large moths (Southwick 1983; Jennersten and Morse 1991). Such cases of mosquito nectar thievery are common among flower visitors that provide no pollinating services (Inouye 1980, 2010; Kevan and Baker 1983; Peach and Gries 2016). The same is true of nectar robbers, which damage flower structures in the process, sometimes allowing the mosquitoes access to the nectar, but again probably without pollinating them.
5. *Alternative Sugar Sources* Attractive features of plants other than their flowers may lead to incorrect conclusions about the mosquito-plant relationship. This includes mosquitoes that may be attracted to the sight or odor of extrafloral nectaries (e.g., Bernath et al. 2016; Sippy et al. 2020; Patt et al. 1999; Gonzalez-Teuber and Heil 2009; Patt and Rohrig 2017; Hoffmeister and Junker 2017) (see *Acacia* and *Lantana* examples below), to honeydew (Peach et al. 2019), to fruits and seed pods (Müller et al. 2010a, b, 2011), to guttation fluids (Urbaneja-Bernat et al. 2020), or to pierced leaves and stems that provide nourishment when nectar is scarce (Müller and Schlein 2005, 2006; Junnila et al. 2010). Extrafloral nectar and honeydew in particular are often overlooked, probably because the sources are difficult to detect (Burkett et al. 1998). Both chemical tests and field observations throughout a season suggest that these may be the dominant forms of sugar sources for mosquitoes in the subtropics and tropics (W. A. Foster et al. unpubl.). In these cases as well, pollination is not involved. The flower connection itself also is often assumed but poorly documented and possibly incorrect. Instead, the implication that mosquitoes are regular flower visitors comes from tests of fructose content. Sometimes these data are persuasive, when based on experimental or circumstantial evidence of attraction, dye uptake, cage feeding, or field correlation between flowering times and sugar contents of mosquitoes.

## Experimental demonstrations of pollination by mosquitoes 

Circumstantial evidence is insufficient to *confirm* pollination. As noted by Adams and Lawson (1993), regarding Australian orchid pollination, “Many scientists in personal discussion confirm that they are aware of the full sequence of pollination and have sometimes seen it, yet have omitted key details in their published reports.” This is a problem with many mosquito-flower studies. Experiments, on the other hand, can sometimes provide strong evidence.

1. Brantjes and Leemans (1976). Pistillate *Silene otites* plants with flower buds were bagged with netting or plastic bags, then a staminate flower stalk was added after the start of anthesis. An insect was added to certain bags during some nights. Later, seed production was assessed. All bags provided with insects yielded good seed production. Two replicates with *Haritata ruralis* (a microlepidopteran moth) produced 146 and 309 seeds*. Xanthorhoe spadaceria* (a geometrid moth) (1 replicate) produced 462 seeds; *Camptogramma bilineata* (a geometrid moth) (3 replicates) produced 131, 36, and 33 seeds; *Euxoa obelisca* (a noctuid moth) (1 replication) produced 453 seeds; and *Cx. pipiens or Culiseta annulata* (mosquitoes) (2 replications) produced 0 and 66 seeds. Unbagged plants produced many more seeds. In a subsequent survey, Dötterl et al. (2012) reported the high pollination potential of *S. otites* visitors during the day instead of night, including 33 species of bees, flies, and beetles in 22 families.
2. Bartareau and Jackes (1994). Flowers of *Pterostylis procera*, a greenhood orchid, were monitored for 3 years, to assess successful pollination both in unencumbered plants and in 28 plants that were enclosed in insect-exclusion bags after being self-pollinated (15) or cross-pollinated (13) by hand. All 100% bagged plants were pollinated, as evidenced by seed-capsule set, whereas unenclosed plants averaged 66.7% when visited naturally (determined by removed pollinium) and 20.7% produced seed capsules. Judging from the mechanism of pollination (a trap-and-release device) and the behavior of the flower visitors in it, the only *confirmed* pollinators would be *Ae. notoscriptus*, *An. annulipes*, and two species of fungus gnats (Mycetophilidae). The experimental and control evidence for mosquito pollination, here, is implicit and indirect. The proportion of visitors performing the required task, mosquitoes or mycetophilids, was not given. Recent studies indicate that mycetophilids are the confirmed pollinators of most *Pterosylis* spp. (Kuiter and Findlater-Smith 2017) (see more below)
3. Peach and Gries (2016). ***Tanacetum vulgare*** (tansy) and three other generalist plants (*Achillea millefolium,* yarrow; *Leucanthemum vulgare,* ox-eye daisy; *Solidago canadensis,* Canada goldenrod) were found by several lines of evidence to be pollinated by *Cx. pipiens*. A few *Cx. tarsalis* and *Culiseta incidens* and 182 *Cx. pipiens* were collected probing tansy flowers in the field during 1-h periods on 5 nights, totaling 5 h of collecting. Furthermore, they acquired tansy pollen grains on their bodies in the laboratory. Self-pollination and cross-pollination experiments were conducted with tansy in laboratory cages, including a mosquito-absent treatment, mosquito-mediated treatments, and blank controls, using two different designs. In the first design, cross- and self-pollination treatments yielded a total of 394 and 99 seeds, respectively, whereas the control treatment yielded only 13 seeds. Mosquito-mediated self-pollinated inflorescences and cross-pollinated inflorescences had mean successful seed-sets per inflorescence of 0.22% and 0.97%, respectively. Control inflorescences with no exposure to mosquitoes had a mean successful seed-set per inflorescence of 0.018%. In the second design (no mosquito-mediated self-pollination treatment), the cross-pollination treatment yielded a total of 3939 seeds, whereas the control treatment yielded a total of only 14 seeds. The mean seed-set per inflorescence for mosquito-mediated cross-pollinated inflorescences was 12.5%, and the control inflorescences 0.06%. Allowing for the limited durations of the experiments, these statistically significant differences are evidence that in the field *Cx. pipiens* might contribute substantially to tansy pollination and seed set. The relative pollination contribution of mosquitoes, as opposed to the many other tansy visitors, is unclear. Aside from the two mosquito species, the following were recorded: common earwigs, goldenrod crab spiders, stink bugs, European honey bees, bumble bees, many other bee species, lygus bugs, ants, nabid bugs, chrysopids, flower flies, other dipterans, vespid wasps, weevils, and lepidopterans. Many of these visitors also probably contribute to pollination.
4. Lahondère et al. (2020)***. Platanthera obtusata*** (the blunt-leafed orchid) is visited naturally only by snow-melt *Aedes* spp. and a few pyralid and geometrid moths. The mechanism of pollination is well understood, and all circumstantial evidence from many studies (including direct observations of the process, the specific action of the volatile blend emanating from the flower, and pollinium counts on insect bodies), indicate that these are the only pollinators in the study areas. Lahondère et al. (2020) has taken the evidence a step further by conducting field experiments. Plants enclosed in insect-exclusion bags had significantly lower fruit-to-flower ratios and seed sets than unbagged plants, and they had elevated fruit ratios in cross-pollinated plants compared to bagged or self-pollinated plants. Field cages containing field-caught mosquitoes and two or more plants had significantly higher fruit-to-flower ratios than bagged plants but were not different than unbagged plants, suggesting that cross-pollination is important. These results confirm the significance of *Aedes* mosquitoes to maximize effective *Platanthera* pollination.

## Synopses of flower-mosquito associations

This section covers the most instructive mosquito studies involving identified flower-bearing plants, possibly with relevance to natural pollination. Tests of mosquitoes that were able to obtain sugar from various plant species when confined with them, such as those of Harada (1971, 1972, 1974, 1975) in Japan, may prove useful when combined with field information. Studies in which sugar contents of field-caught mosquitoes were reported but the sugar sources were unknown are included only for the two disease vectors, discussed below. The following information on plant-visiting mosquitoes is based on representative studies and observations made in North America, Central America, Europe, the Middle East, Africa, Southeast Asia, and Australia. Although categorized as specialists or generalists, these are not neat distinctions. It will be evident that the great majority of these flower associations appear to involve generalist (promiscuous) plants visited by generalist (promiscuous) mosquitoes, and therefore a few are mentioned twice. The diversity of alternative pollinators also is presented. The two disease vectors will be reviewed separately (Section V, below).

### A. Selective associates

*1) Selective Plants.* The snow-melt mosquitoes *Ae. communis, Ae. nigripes, Ae. canadensis*, *Ae. increpitus*, and other snow-melt *Aedes* spp of northern North America typically visit primarily flowers of *P. obtusata*. Mosquitoes bearing *P. obtusata* pollinia are common, and the method of attachment and subsequent detachment of the orchid’s pollinia have been described in detail (Hocking et al. 1950; Stoutamire 1968; Thien 1969a, b; Thien and Utech 1970; Goreham 1976; Voss and Riefner 1983). Unlike others, the snow-melt *Aedes* spp. appear to be adept at entering the blossoms quickly and obtaining its nectar while removing or placing the pollinia (Thien 1969b). The blend of volatile chemicals released by *P. obtusata* flowers, which are dominated by nonanal but with lilac aldehyde as an important minor component, attracted primarily *Ae. communis* and *Ae. increpitus* and a few other *Aedes* spp. (Lahondère et al. 2020). *Ae. communis* appears to be the most common pollinator, though pyralid and geometrid moths also may play substantial roles. Thien and Utech (1970) found bog orchid pollinia on 45% of two species of geometrid moths in northern Wisconsin, in addition to those on *Ae. communis* and a few other *Aedes* spp. The moths also carried many more pollinia per insect, usually 2.5 times as many as the mosquitoes, and up to 14. They concluded that moths also should be considered as important pollinators of *P. obtusata*. Moths may be more efficient and effective pollinators at times, either because of the greater length of their proboscises or the less dense mosquito populations at the southern limits of the bog orchid’s range. Lahondère et al. (2020) reported that they saw a moth at a *P. obtusata* flower only once in Washington, USA.

As noted by Thien and Utech (1970), *Ae. communis* and other pollinating species may visit alternative flowers or have alternative sources of sugar and therefore may switch between *P. obtusata* and other blooming plant species, according to seasonal availability or locality, and are not dependent on it. Furthermore, *Ae. communis* thrives in many localities where *P. obtusata* does not exist and feeds on the flowers of common plants in the USA (Grimstad and DeFoliart 1974) and in Sweden (Andersson 1990). The same is true for some of the pyralid moths that are apparent *P. obtusata* pollinators in Michigan but whose range extends well south of the orchid’s limit (Voss and Riefner 1983). Thus, this plant may depend on one or a few species of mosquitoes and moths, but not necessarily vice versa. It should be noted that *Ae. communis* is considered to be a complex of four mosquito species in mountainous western North America and in central Canada (Brust and Munstermann 1992), and perhaps more in other regions, including Eurasia (Schutz and Eldridge 1993). These and other cryptic species may differ in their attraction to various nectar sources.

*P. procera*, a greenhood orchid widespread in Australia, appears to be another specialist, originally reported to be pollinated only by the mosquitoes *Ae. notoscriptus* and *An. annulipes*, and two species of mycetophilid fungus gnats (Bartareau and Jackes 1994). Studies of other Australian *Pterostylis* spp. indicate that *Culex* spp., *An. farauti*, and mycetophilids also are *probable* pollinators, based on the presence of a pollinium on the thorax (Adams and Lawson 1993; Bartareau and Jackes 1994). Recent investigations of several *Pterostylis* spp. in Australia and New Zealand (see Phillips et al. 2014; Bodley et al. 2016; Reiter et al 2019; Hayashi et al. 2021) have investigated their pollination systems in greater detail and indicate that male mycetophilid and keroplatid fungus gnats are the principal pollinators, which enter a trap-like device where pollinia are attached to the insects’ thorax. Their odors serve as sex attractants to males, an example of pollination by way of plant–insect exploitation by deception. The reason for the attractiveness to female mosquitoes, which are not known to utilize volatile sex pheromones, remains to be investigated.

*2) Selective Mosquitoes.* The mosquito *Ae. provocans* feeds in large numbers, by default, only on *Prunus pensylvannica* (pin cherry) and *P. nigra* (Canada plum) blossoms in Ontario, Canada, when flowering and mosquito emergence coincide early each year (Gadawski and Smith 1992). However, both the difficulty of *Ae. provocans* in entering the *Prunus* flowers and its restricted movement between flowers appear to minimize opportunities for pollination (Smith and Gadawski 1994). Several genera of native bees (Hall et al. 1981), among other kinds of insects, start visiting the *Prunus* flowers after the main wave of emergence of mosquitoes has ended and may play a larger part in pollination.

### B. Two-way promiscuity of associates

*Clematis lingusticifolia* flowers were visited by unidentified mosquitoes in Alberta, Canada, and made up less than 7% of all visitors (Borkent and Harder 2007). Several species of large and small muscoid flies made up 42% and 33% of flower visitors, respectively. The next most abundant visitors were halictid bees (10%). Muscoid fly visitation frequency (number of individuals per branch of flowers per time period) did not vary from that of halictids and mosquitoes, but mosquitoes visited substantially fewer flowers per second than halictids did. Larger visitors tended to visit flowers more quickly than smaller visitors. There was no detected difference in the number of visits to the same flower among any of the insect groups.

*Silene otites* flowers are frequently visited at night by the mosquitoes *Cx. pipiens* and *Cs. annulata* and various moths in these groups: Microlepidoptera (5 spp), Plutellidae (1 sp.), Pterophoridae (1 sp.), Geometridae (26 spp.), Noctuidae (16 spp.), as well calliphorid flies and a neuropteran (Brantjes and Leemans 1976). Though the mosquitoes and moths were considered to be its main pollinators, more recently Dötterl et al. (2012) have found that diurnal visitors, including bees, flies, and beetles, may be pollinators as well.

In arctic North America, the snow-melt mosquitoes *Ae. impiger* and* Ae. nigripes* visit the flowers of *Rubus chamaemorus, Platanthera hyperborea, A. millefolium, P. obtusata, Salix arctica,* and *Dryas integrifolia.* They also are visited by species of the following orders and families of insects: the Lepidoptera: Noctuidae, Lycaenidae, Pieridae, Nymphalidae; the Diptera: Chironomidae, Muscidae, Calliphoridae, Scatophagidae; and the Hymenoptera: Bombidae, Apidae (e.g., Hocking (1953, 1968).

*Cx. pipiens*, *Cx. tarsalis,* and *Cs. incidens* visited the generalist invasive *Tanacetum vulgare* (tansy) flowers in British Columbia, Canada, and also *A. millefolium*. Other tansy flower visitors were a diversity of flies, bees, wasps, ants, lepidopterans, beetles, hemipterans, lacewings, and earwigs (Peach and Gries 2016). In Europe, the main visitors of tansy are solitary bees, flies, and beetles (e.g., Dupont et al. 2018, Willaert 2019, Balkan Ecology Project: https://www.balkep.org › tanacetum-vulgare). In parts of Sweden, *Culex* spp., *Aedes* spp., and *An. messae* are common tansy and yarrow flower visitors (Andersson and Jaenson 1987; Andersson 1990; Jaenson and Ameneshewa 1991).

*Ae. taeniorhynchus* (the black salt-marsh mosquito) feeds on the flowers of salt-marsh plants (*Avicennia germinans* (= *A. nitida*) (black mangrove) and *Conocarpus erectus* (buttonwood), but also on *Eupatorium* sp. and *Bidens* sp. It occurs on the blossoms of *A. germinans* and *C. erectus* in prodigious numbers when the mosquitoes emerge synchronously following high tides and heavy rainfall that stimulate egg hatching in coastal Florida, USA. Other sources of sugar are aphid honeydew and extrafloral nectaries of buttonwood, which ranked second and third in importance compared to the flowers of the salt-marsh plants (Haeger 1955, 1960). Pollination by the mosquitoes seems likely to occur at these times. Visits of *Ae. taeniorhynchus* to the flowers of other species at eastern coastal locations throughout the Americas have not been investigated. Unspecialized pollinators, primarily bees, wasps, flies, butterflies, and moths, appear to be important most of the time (Lonard et al. 2017, 2021).

In the tundra of arctic Canada, *Aedes* spp. mosquitoes visited flowers of *Pedicularis capitata* (capitate lousewort)*, P. arctica* (arctic lousewort)*, S. arctica* (arctic willow)*, D. integrifolia* (entire-leaf mountain-avens)*,* and *Saxifraga oppositifolia* (purple saxifrage). Medium and large-bodied Diptera and bumble bees were the other insects commonly visiting these flowers (Kevan 1972). Of those four plant species, mosquitoes were considered to be among the pollinators only of *D. integrifolia* and were of "lesser importance" than the large bees and flies. In Greenland, despite the abundance and species richness of insects in the high arctic, by their sheer numbers muscid flies—not mosquitoes—are considered to be the “…key predictors of *D. integrifolia* seed set” (Tiusanen et al. 2016).

Mosquitoes in the genera *Aedes* (10 species), *Coquillettidia* (1 species), *Culex* (2 species), and *Culiseta* (1 species) visited the flowers of many of the 39 species in the plant families Asteraceae, Asclepiadaceae, Rosaceae, Apocynaceae, Umbelliferae, Oleaceae, among others recorded in a study area in Minnesota, USA (Sandholm & Price 1962). Many of these plants are known to be visited frequently by many other insect species in several orders (W.A. Foster unpubl.). Likewise, in Wisconsin, USA, 23 mosquito species in 5 genera visited *Achillea millefolium* (yarrow), *Asclepius syriaca* (common milkweed), *Leucanthemum vulgare* (ox-eye daisy), *Cornus stolonifera* (red-osier dogwood), *and Solidago* spp. (goldenrod) (Grimstad and DeFoliart 1974). Other plant species were visited by mosquitoes less often. Some mosquito species occurred more frequently on some plant species than on others, independently of their relative availability or the stage of their blooms, indicating a selective bias. Other insect visitors were not mentioned.

Similarly, the saltmarsh mosquitoes *Ae. sollicitans and Ae. cantator* in coastal Connecticut, USA, visited *Taraxicum officinale* (common dandelion), *Rosa palustris* (swamp rose*), A. millefolium*, *C. vulgare Daucus carota* (Queen Anne's lace), *Malva neglecta* (common mallow), *Hieracium pratense* (king-devil hawkweed), *Gerardia maritima* (seaside gerardia), and *Solidago sempervirens* (seaside goldenrod). Their flower prevalence shifted as the blooming season progressed (Magnarelli 1977, 1978). Likewise, in inland Connecticut, USA, the woodland mosquitoes *Ae. canadensis* and *Ae. stimulans* frequented *A. millefolium*, *Actaea pachypoda* (white baneberry), *Brassica rapa* (bird’s rape), *Lychnis alba* (evening lychnis), *Maianthemum canadense* (wild lily-of-the-valley), *Smilacina racemosa* (false Solomon’s seal), and *Viburnum acerfolium* (maple-leaf viburnum). Their predominating presence on flowers of a plant species shifted with the season of maximum blooming (Magnarelli 1983). The timing of other insect visitors was not mentioned.

In Ohio, USA, the mosquitoes *Ae. vexans, Ae. trivittatus,* and *Culex spp.* visited flowers of *D. carota*,, *A. syriaca, Apocynum medium* (intermediate dogbane), and * L. vulgare* (Yee et al. 1992). *A. syriaca* generally was the plant most frequently visited, but its floral structure precludes pollination by mosquitoes (Jennersten and Morse 1991). The others have an open structure typical of generalist flowers. The flowers of all four plants were commonly visited by multiple species of several orders of insects (e.g., moths, butterflies, flies, beetles, wasps, and bees), both day and night (W.A. Foster unpubl.).

Mosquito species of the genera *Aedes, Anopheles, Culex, Deinocerites, Toxorhynchites,* and *Mansonia* visited the flowers of *Terminalia catappa* (Indian almond) and *Clibadium glomeratum* in Costa Rica (Foster, W.A. unpubl.). However, most were feeding on the extrafloral nectaries (EFNs) of *T. catappa*, *Hibiscus tiliaceus (*beach hibiscus), *Ochroma lagopus* (balsa)*, Heliconia latispatha* (expanded lobsterclaw), *Costus* sp. (ginger), and on hemipteran-punctured fruit of *Lycianthes maxonii* (Solanaceae). In one study of *T. catappa* in India, the flower visitors included native bees, ants, wasps, flies, and butterflies, altogether 17 insect species (Alturi et al. 2003). Bees in the genera *Apis* and *Trigona* and flies in the genera *Chrysomyia* and *Sarcophaga* were considered to be the important pollinators, by collation of three factors: frequency of visits, foraging speed, and pollen pick-up.

In forests of eastern Panama, mosquito species of *Aedes, Haemagogus, Psorophora, Culex, Mansonia, Wyeomyia,* and *Toxorhynchites* visited flowers of *Erythroxylum* cf. *havanense* and *Erythroxylum* sp. aff. *lucidum* during their brief blooming season, as well as large numbers of bees, wasps, and beetles. In open disturbed areas of central and eastern Panama, mosquitoes visited the EFNs of *O. lagopus, H. latispatha, Tetracera volubilis, Anacardium occidentale* (cashew)*,* and *Persea americana* (avocado). These supply sugar for long periods and dilute the potential pollination contribution of mosquitoes. *Aedeomyia squamipennis, Cx. erraticus/ocossa, Anopheles spp.,* and *Mansonia dyari* were the dominant species on cashew EFNs. These mosquitoes fed only on the EFNs of cashew’s floral and foliar panicles (Wunnachit et al. 1992) and ignored the flowers themselves (W.A. Foster unpubl.). Detailed studies elsewhere indicate that 40 insect species visit cashew flowers, but the only visitors considered to be pollinators in one Indian locality, for example, are 13 bee species: eight Apidae and five Halictidae (Vanitha and Raviprasad 2019). Mosquitoes also were seen only on the EFNs of avocado, not on its flowers. In other studies, avocado pollination is reported to be achieved by bees, wasps, moths, butterflies, and flies (Buxton et al. 2021). Whether mosquitoes visit cashew and avocado flowers in other localities or other seasons has not been reported.

## Evaluation of mosquito pollination in general

The arguments presented below address the points relevant to an evaluation of mosquito pollination. These are, however, simplifications of the many factors needed to establish their precise role in specific cases. In some instances, the evidence provides enough information to surmise a mosquito’s *possible* or *probable* status as a pollinator in the Adams-Lawson (1993) sense. But few studies of *probable* or even *confirmed* pollinators allow speculation about the size of their contribution (i.e., *important, minor, trivial,* or *redundant*) to a plant’s reproduction; usually they are based only on guesses of their *efficiency* and *effectiveness*. The most prominent missing pieces of information are these: (1) There is no precise description of the behavior of the mosquitoes at the flower(s) in the field or laboratory. (2) There is either no knowledge or incomplete knowledge of the frequency of visits to the flowers in comparison to the flowers of other plant species, or to non-floral sugar sources in the field. (3) The alternative potential pollinators of the plant(s) in question, and their abundance and the frequency of their visits, are almost never mentioned. Studies that include either multiple species of mosquitoes or multiple species of plants, in the few cases where they are mentioned, make the assessment even more challenging. (4) Experiments and observations comparing behavioral pollination traits of mosquitoes and alternative flower visitors, either in the field or in cages, are scarce.

The following section briefly summarizes the mosquito-flower studies cited above according to information that either supports or detracts from the hypothesis that mosquitoes in general are likely pollinators.

### A. Supports – for the hypothesis that some mosquitoes are important pollinators

1. *Pollen Transport*. Some species collected in the field carry pollen grains on their bodies and extremities or carry pollinia attached to their eyes. For snow-melt mosquitoes, both the removal of pollinia from anthers of the specialized flowers of the bog orchid *P. obtusata* and their attachment to the stigmas of other *P. obtusata* flowers have been observed directly. Furthermore, only a small guild of snow-melt *Aedes* species and a few moths are adept at accessing the nectar of that orchid and responding to its particular odor blend. The flowers of *P. obtusata* and the semi-specialist plant *P. procera* are exceptional in exhibiting narrowly composed mechanisms for pollen pick-up and deposition between flowers by their primary or exclusive mosquito or fungus gnat visitors.
2. *Experimental Pollination*. Laboratory experiments demonstrated that *Cx. pipiens* can effectively cross pollinate between individual plants of the generalist flowers of tansy. Field experiments with bagged and unbagged flowers demonstrated that maximum pollination was achieved when insects had access to flowers. Much higher seed-set of *Silene otites* flowers occurred when they were not bagged and therefore did not exclude mosquito and moth potential pollinators. Bagging experiments with *P. obtusata* likewise demonstrated the importance of *Aedes* spp. visits to maximize fruit formation and seed-set. Bagging experiments with *P. procera* also demonstrated pollination by the combined activities of its presumptive pollinators: two mosquito species and two fungus gnats.
3. *Mosquito-Plant Fidelity*. Aside from the exceptional cases of the specialist visitors to *Platanthera* and *Pterostylis spp.*, nearly all mosquito species appear to be generalists. They do, however, show a range of preferences owing to differences in innate flower attractiveness (e.g., Yu et al. 2016, 2018), which results in a degree of flower constancy. That attraction may be an adaptation due to the nutritional benefits of flower nectar, which contains not only sugar but several minor ingredients, notably amino acids and vitamins. These ingredients prolong survival and contribute to reproduction. To some extent flower constancy is maintained by availability and perhaps learning, because the flower visits are focused on the species in bloom at particular parts of the year or those that are aggregated in particular locations.
4. *Mosquito Abundance.* The presence of very large numbers of mosquito visitors to the flowers of some plants probably promotes pollination, though direct evidence is lacking. This situation occurs either seasonally or under specific environmental conditions, such as when plant blooming coincides with periods or locations of exceptional mosquito abundance. Impressive examples are these: a) the massive emergences of *Ae. taeniorhynchus*, whose adults feed on the blossoms of the mangroves; b) the great variety of mosquitoes and other flower visitors that emerge and feed on the blossoms of *Erythroxylum* spp. at the beginning of the rainy season in Panama; c) *Ae. caspius* and *An. sergentii* that develop at the oases in the semi-desert of the Jordan Valley, Israel, and feed on the flowers of the xeric plants *Tamarix nilotica* (Nile tamarisk) and *Ochradenus baccatus* (Resedaceae) that bloom there during the dry season (Müller and Schlein 2006); and d) the blooms of tundra plants and emerging snow-melt *Aedes* spp. that feed on them during the short summer in arctic Canada and Greenland.

### B. Detractions–weakening the hypothesis that some mosquitoes are important pollinators

1. *Mosquito and Plant Promiscuity.* Mosquitoes tend to be promiscuous (generalists), visiting a wide variety of plant species. There appear to be no pivotal mosquito species, but rather a broad diversity of them among several genera, perhaps uselessly spreading pollen among a variety of unrelated plants. Flower constancy is rare, except as enforced by distribution and seasonality of different host plants (see above). Also, the plant species they visit are typically promiscuous, their flowers being visited by a wide variety of other insects in several insect orders that may serve as pollinators. Specialist relationships such as the orchids *P. obtusata* and possibly *P. procera* are the exceptions. Flowers of the orchid *Malaxis monophyllos* appear to be visited only by small Diptera, including at least five families, one of which is Culicidae (Jermakowicz et al. 2022). A similar lack of mosquito-plant specificity has been recorded for many other uncertain relationships, including those in Southeast Asia (e.g., Kato 1996).
2. *Behavioral Inefficiency.* As observed by Inouye (2010), mosquitoes that visit flowers are poorly suited for the pickup and deposition of pollen during nectar feeding, and therefore are not prominent pollinator candidates. Some mosquito researchers have agreed that their behavior contributes but is not conducive to effective and efficient pollination. Examples: (a) Based only on numbers collected on flowers and pollen on insect bodies, Kevan (1972) determined that mosquitoes were among the insects of “lesser importance” as pollinators of *Dryas integrifolia* on the tundra, but nevertheless were necessary for full seed set. Primary pollinators were large Diptera and bumble bees, two kinds of insects that can best be described as strong, energetic, and boisterous. (b) Brantjes and Leemans (1976) concluded that mosquitoes moved only infrequently between flower stalks, so their potential for pollination, per mosquito, would not be large. They surmised that mosquitoes were the *primary* pollinators of *Silene otites* only when they were particularly numerous and moths were impeded by bad weather. Otherwise, moths were the main pollinators. A different conclusion might have been reached if the contribution of diverse insects that visit the flowers during the day (Dötterl et al. 2012) had been estimated. (c) Smith and Gadawski (1994) suggested that *Aedes provocans* is probably a mediocre pollinator of *Prunus* spp., despite being a specialist by default, due to the coincidence of mosquito emergence and flower availability. Its foraging was inefficient, and inter-flower dispersal was limited. A mosquito frequently returned to feed on a flower it had left only minutes earlier, and flights to adjacent blossoms were rare, averaging about every 40 min. They seldom carried pollen grains, and details of their flower handling were such that they did not contact the pistil. (d) A study of *Clematis lingusticifolia* flower visitors found that a great majority (75%) were muscoid flies (Borkent and Harder 2007). Muscoid fly visitation frequency (number of individuals per branch of flowers per time period) did not differ significantly from that of halictid bees and mosquitoes, but mosquitoes visited significantly fewer flowers per second than halictids and large muscoid flies. Those large flies tended to visit flowers more quickly than smaller visitors. Small muscoid flies visited flowers at the same rate as mosquitoes.
  Even casual observations give the impression that diurnal, crepuscular, and night-feeding mosquitoes all spend much more time probing and exploring generalist flowers than most other visitors. This seems to hold true for bees, wasps, butterflies, and large flies during the day, and small and medium-sized moths (noctuids, geometrids, large pyralids) during the night. In particular, bees of many families tend to be fast at locating and withdrawing nectar from a variety of flowers and to wallow or use “buzz pollination” among anthers to gather large amounts of pollen on their bodies. They move quickly among separate flowers and between different blooming plants, and some of them have special tactics to minimize wasted time, such as using memory to select productive species or an instinctive foraging pattern to transition between flowers to avoid repeated visits. Thus, it appears that body size, speed, and efficient flower-handling methods all favor pollination by insects other than mosquitoes and their delicate dipteran relatives.
3. ***Alternative Generalists.*** A great variety of generalist insects, especially among bees, wasps, beetles, butterflies, moths, and flies, are listed in the literature as being common visitors and probable pollinators of the flowers that mosquitoes also are known to visit. As indicated above, most of these flower visitors have characteristics that make them more effective and efficient, including flower-handling speed, pollen-collecting devices, high vagility among different flowers and different plants, and medium-to-large body size. Thus there may be local competition among plants for all potential generalist pollinators.
4. ***Low Representation.*** Various insects listed in the literature as probable pollinators of mosquito-visited plants very rarely include mosquitoes among them. Surveys of flower visitors typically include a wide variety of alternate generalist insects, including various orders and species, but mosquitoes seldom or never. An exception is the direct observational study of Borkent and Harder (2007), which included all alternate visitors in their survey and found that only 7% of the visitors to *Clematis* blooms were mosquitoes. A strong correlation between numbers of mosquitoes at flowers and their density in the local population is unlikely, because they often obtain sugar from non-floral sources such as extra-floral nectaries and honeydew, depending on the local habitat. A second confounding factor is the females' use of human blood as an exceptionally large source of energy as an alternative to plant sugar, particularly among the top disease vectors (see below).
5. ***Weak Experimental Evidence.*** In lab experiments, pollination of tansy by *Cx. pipiens* appears to be weak or inefficient, judging from the very low resulting seed-set in plants caged with mosquitoes (Peach and Gries 2016). By one measure, the mean seed-set per inflorescence for mosquito-mediated cross-pollinated inflorescences was only 12.5%, which was nevertheless significantly higher than the 0.06 seed set of the controls. The small proportions may be a result of the time-limit of the experiment. A 3-year field study of *P. procera* gave a broader perspective. Manually-pollinated bagged flowers, used as experimental controls, gave 100% seed-capsule set, whereas among flowers exposed to insects naturally only 2/3 had their pollinia removed, and only 21% produced seed capsules (Bartareau and Jackes 1994). The authors concluded that about three flower visits were required for maximum pollination. The result suggests that local mosquitoes and fungus gnats achieved only moderate pollination effectiveness, perhaps due to their low numbers. In addition, flower visits by mosquitoes might be reduced by the absence of a reinforcing payoff to boost *P. procera*'s repeated attractiveness to mosquitoes—if indeed the semiochemicals released by the orchid serve both as resource kairomones for mosquitoes and as sex pheromones for the gnats (Hayashi et al. 2021). As yet, there is no evidence for this dual system.

### C. Summary and conclusion

The evidence presented above makes clear that some mosquitoes are probable pollinators of some plants. The question, then, is whether their contribution to this service is biologically relevant. For the great majority of mosquito-plant associations studied so far, the contribution of mosquitoes in general—or any particular mosquito species—appears to be minor, trivial, or redundant. The mosquito-pollination hypothesis is greatly weakened by the well documented multitude and diversity of other insects that visit the same plant species, many of which have superior pollinating characteristics. Another factor weakening that hypothesis is the diversity of plant species that each mosquito species visits.

## Principal mosquito disease vectors

The observations and conclusions presented above about mosquito pollination in general apply equally to the two principal vectors of human disease. The following overview also takes a hypothetical approach, in this case the possibility that *An. gambiae* (which transmits malaria and filarial parasites) and the domestic form of *Ae. aegypti* (which transmits yellow fever, dengue, Zika, and Chikungunya arboviruses) have characteristics that may either support or diminish their prospects as possible pollinators. These species are the focus, here, for two reasons: 1) They have an especially devastating effect on the health of human communities, and 2) their concentration around human habitations makes them suitable or accessible targets for local elimination by either conventional or molecular-genetic methods. Vector population suppression or elimination might be unwarranted if its downstream effects on the structure of biotic communities interferes with pollination systems. This topic is speculative because we know so little about the flower-visiting behavior of these two species.

### A. Vector characteristics that diminish flower visits:

*1) Low Sugar-Feeding Frequency. An. gambiae* and *Ae. aegypti* feed on sugar less often than other mosquitoes because the females are adapted for human blood-feeding. In both temperate and tropical regions, many Anophelines and the Aedines in the subgenus *Stegomyia* (including *Ae. aegypti* and *Ae. albopictus*) are among the mosquitoes least likely to carry recent sugar meals (e.g., Bidlingmayer and Hem 1973; Edman et al. 1992; Beier 1996; Martinez-Ibarra et al. 1997; Costero et al. 1998; Burkett et al. 1999) and least likely to be observed on flowers or extrafloral nectaries (e.g., Sandholm and Price 1962; Grimstad and DeFoliart 1974; A.W.R. McCrae pers. comm., W.A. Foster unpubl.). This seems to be true both in Africa and in Central and South America (W.A. Foster 2022, H. Manda et al. unpubl.), though recent studies on those two *Stegomyia* species suggest sugar feeding is sometimes more common than previously thought (Sissoko et al. 2019; Olson et al. 2020; Swan et al. 2021; Wanjiku et al. 2021). These different results may be caused by differences in environment, season, and availability of blood hosts (Van Handel et al. 1994; Costero et al. 1998; Spencer et al. 2005; Fikrig et al. 2020; Olson et al. 2020; Swan et al. 2021).

Infrequent sugar feeding by these strongly anthropophilc species also may have physical and physiological bases. *An. gambiae* appears to ingest relatively small replete sugar meals, owing to the limited expandability of its abdomen, and it digests its sugar meals much more quickly than culicine mosquitoes (Gary 2005). Females of both vectors convert an unusually large portion of each human blood meal into stored energy (Briegel 1985; Fernandes and Briegel 2005) and forgo some plant-sugar meals. One consequence is that they often ingest blood frequently, despite the metabolic cost of a high-protein and low-carbohydrate diet, which shortens life but increases egg production per unit of time (Braks et al. 2006; Simpson and Raubenheimer 2009). This circumstance may result in fewer visits to sugar sources, including flowers, and thus fewer opportunities for pollination.

*2) Alternative Sugar Sources.* In the tropics these and other mosquito species appear to obtain sugar primarily from non-floral sources, including extrafloral nectaries, hemipteran honeydew, damaged or rotting fruit, and phloem sap from plant tissues. Field observations suggest (A.W.R. McCrae pers. comm.) persuasively that most mosquitoes in sub-Saharan Africa use extrafloral nectar, and perhaps honeydew and damaged leaves and fruit, rather than flower nectar. This view has been reinforced (W.A. Foster et al. unpubl.) through direct field observations of *An. gambiae* s.l. and other mosquito species at night in western Kenya, and also in cage and mesocosm experiments with invasive and native Kenyan plants. Those studies indicated that extrafloral nectaries and honeydew were their main sugar sources (Stone et al. 2012; Ebrahimi et al. 2017; W.A. Foster et al. unpubl.), which are not connected to pollination. Only on *Parthenium hysterophorus*, the strongly attractive invasive weed from South America, has *An. gambiae* been seen probing its flowers in the field (W.A. Foster et al. unpubl.). Laboratory studies, fructose tests of field-captured specimens, and tentative bar-code identifications of plant DNA in *An. gambiae* and *Ae. aegypti* (Nyasembe et al. 2018; Sissoko et al. 2019; Wanjiku et al. 2021) confirm that they feed on plant sugar in the field, perhaps sometimes from flowers. Firm evidence that they might serve as occasional pollinators remains elusive.

An exception could be the ubiquitous *Lantana camara* (lantana), whose flowers are considered to be only weakly attractive (Senior White 1952; McCrae et al. 1968; Manda et al. 2007a, b; Gouagna et al. 2010, 2014; Sissoko et al. 2019) or even repellent (Dua, et al. 1996; Mng’ong’o et al. 2011), but whose florets mosquitoes will probe in the field, despite appearing to be unable to reach the nectar (W.A. Foster unpubl). Butterflies, bees, and hummingbirds are considered to be among its main pollinators (e.g., Maharaj and Bourne 2017). Instead, *L. camara*’s foliar nectaries (EFNs) in the calyces beneath the inflorescences—not their flowers, per se—may be the source of their sugar (Inamdar 1969; Nikbakhtzadeh et al. 2016; W.A. Foster unpubl.), making them unlikely pollinators of *L. camara*.

*3) Low Population Densities.* The abundance of these vectors is often lower than mosquitoes in general. With few exceptions, the mosquitoes known to visit African flowers or tropical or subtropical American flowers in large numbers are not *Anopheles* malaria vectors or prominent *Aedes* arbovirus vectors, but rather a wide assortment of other culicine species. In Panama, Costa Rica, and western Kenya, Anophelines and the Culicines in the subgenus *Stegomyia* (e.g., *Ae. aegypti* and *Ae. albopictus*) constitute a small minority of mosquito species visiting plants (W.A. Foster unpubl.). The same seems to hold true in Uganda and The Gambia (McCrae et al. 1968 and pers. comm.), cf., *An. implexus* on *Harungana madagascariensis* flowers (McCrae et al. 1976), possibly simply because anopheline population densities typically are low—even in areas of moderately high malaria transmission (e.g., N’Guessin et al. 2014; Simsek 2006).

*4) Energy from Human Blood.* This characteristic is tied to the first reason, i.e., the females derive more energy from human blood and less from plants (see Introduction, above, and Foster 2022). This appears to be an adaptation to human-altered landscapes that offer a) an abundance of artificial aquatic sites for larval development, b) artificial resting sites, protecting the adults from harsh conditions during periods of inactivity, and c) easy access to humans for blood. These landscape features typically are disturbed habitats lacking native plants—though some invasive plants and ornamental and agricultural species can provide sugar. Human-altered settings are especially amendable to invasive mosquitoes such as *Ae. aegypti*, which has spread from sylvan African settings into villages and then into urban areas, and now has colonized most tropical and subtropical areas of the world. *An. gambiae* likewise probably became adapted to large numbers of human-altered habitats in Africa, offering fewer natural sugar sources but high aggregations of energy-rich blood sources and larval sites.

### B. Vector-flower case studies

*1) Aedes aegypti.* This section expands on the domestic form of *Ae. aegypti*, the notorious pantropical and subtropical vector of urban yellow fever, dengue, Zika, and Chikungunya viruses. The incomplete information on their flower associations will be reviewed here. That information includes evidence of sugar feeding, based on attraction to flowers or cuttings bearing flowers, plant-sugar contents of collected mosquitoes, and presence of plant DNA in the mosquitoes. In field studies where flower visits have been established, direct observations and information on the frequency of other visitors to those flowers is lacking. Therefore, a comprehensive picture of the relative importance of all natural sources of sugar to *Ae. aegypti*, and vice versa, is incomplete.

*Ae. aegypti* females collected in different urban zones of Huixtla, southern Mexico, by Martinez-Ibarra et al. (1997) were tested for fructose. The results were compared to the corresponding presence of particular flowering plants in each zone. This information was used to infer possible host plants. Females collected in houses in the outskirts of the city more often were fructose-positive than in more centralized zones, and the outskirts had the greatest number of flowering plants per household. Fructose positivity was highest in houses with the ornamental plants *Bougainvillea* spp., *Hibiscus rosa-sinensis*, and *Rondeletia* spp., and in houses associated with the shrubs *Ludwigia octovalvis*, *L. camara*, *Sida acuta*, and *Bidens* spp. Other studies have recorded the prevalence of fructose in *Ae. aegypti* in various regions of the world, and its variation with season, environment, and sex: Puerto Rico (Costero et al. 1998), Thailand (Spencer et al. 2005), Kenya: (Nyasembe et al. 2018), and Texas, USA (Olson et al. 2020). But these studies have not implicated particular plant species as sources of their sugar.

In three locations in western Kenya, Nyasembe et al. (2018) reported indirect evidence of *Ae. aegypti* feeding on *Hibiscus heterophyllus, Senna uniflora,* and *Pithecellobium dulce.* Plant-host identifications were based on matches of nucleotide sequences of targeted genes (“bar-coding”) of plant chloroplast DNA with those contained within fructose-positive samples of field-caught mosquitoes. *Hibiscus heterophyllus* is an Australian native pollinated by birds, butterflies, moths, and bees (https://www.nparks.gov.sg/florafaunaweb/flora/5/5/5541). *Senna uniflora* probably is pollinated by large bees (Marazzi and Endress 2008), and *P. dulce* so far is known to be visited only by birds, moths, butterflies, and other heavy-bodied insects (e.g., Torres Gonzalez et al. 2014). Direct observations of floral visits by *Ae. aegypti* to these plants have not been reported. The first two plants have close relatives with extrafloral nectaries utilized by other mosquito species (field observations: W.A. Foster unpubl.). Thus, non-floral plant sources of the DNA are possible. The presence of many alternative flower visitors to these plant species, their likely ability to cross-contaminate flowers of different species, and the possible alternative sources of sugar for the mosquitoes, all indicate that their pollination by *Ae. aegypti* is likely to be small.

In Bamako, Mali, Sissoko et al. (2019) reported evidence of *Ae. aegypti* feeding on the flowers of *Acacia macrostachya, Prosopis juliflora, Acacia salicina, Galphimia gracilis, Mangifera indica* (mango)*,* and *Ricinus communis* (castor bean). Attractiveness was measured by catch size of field traps baited with specimens of 20 plant species: flowers, seed pods, honeydew, or fruit. The most attractive plants in flower were *A. macrostachya, P. juliflora, A. salicina,* and *G. gracilis.* In cages, the blossoming plants from which they most readily ingested nectar (as demonstrated by fructose contents) *were P. juliflora, A. macrostachya, A. salicina, M. indica, R. communis* (including its petiolar extrafloral nectaries), and *G. gracilis*. Among those six plants tested for promoting mosquito survival in a cage, *P. juliflora* was best and *G. gracilis* was good. Fruits and honeydew also were attractive and induced feeding but scored much lower than the best flowers. There were no reports of direct observations of plant-feeding behavior of any kind, either in the field or in cages. Tests for fructose in field-caught mosquitoes and cage exposures indicated, however, that floral nectar feeding might be common. Thus, pollination is possible for the proposed plant hosts tested, with the likely exception of *R. communis* (see Nyasembe et al. 2018 comment, below). However, the great variety of insects known to visit the kinds of flowers studied here (see, e.g., Gouagna et al. 2010, Montesinos et al. 2016, Müller et al. 2017, above) indicates that the pollinating contribution of *Ae. aegypti* is likely to be quite small or redundant.

Direct field observations overnight on *A. macrostachya* during its flowering season in The Gambia revealed that all feeding by mosquitoes of several species, including *An. gambiae* and *Ae. aegypti*, occurred on EFNs of the acacia’s leaf petioles, not on its closely associated flowers (A.W.R. McCrae pers. comm.). If confirmed, one may conclude that despite *A. macrostachya’*s great attractiveness to mosquitoes, they may play little or no role in its pollination. (A similar situation appears to be true for *Anacardium occidentale* in Panama; see above). In Kilifi and Isolo, Kenya, Wanjiku et al. (2021) detected evidence of *Ae. aegypti* feeding on 34 plant species belonging to 21 families, based on a DNA bar-coding technique similar to that of Nyasembe et al. (2018). Of those 34 plant species, only 7 were found growing at the study sites: *P. dulce*, *Zea mays* (corn or maize), *Vigna unguiculata*, *Hibiscus* spp., *Malva parviflora*, *Acacia* spp., and *Azadirachta indica* (neem tree). Several of these appear unlikely to have been derived by floral nectar-feeding, given their flowers’s structure and known or likely pollinators (W.A. Foster unpubl.). *P. dulce* may be an exception but appears to have many flower visitors (see Nyasembe et al. 2018, above), so the mosquito contribution to its pollination may be small. No direct observations were made. Wanjiku et al. (2021) inferred that some of the DNA samples may have come from pierced plant tissues. Contamination by other plant species, via polylectic insects visiting a series of different flowers, or DNA from honeydew or EFNs, also could account for the wide assortment of plant DNA identified.

*Summary: Ae. aegypti* is a confirmed feeder on plant sugar, and some of that sugar may come from flower nectar. Very few studies support this speculation, however, and none excludes honeydew, extrafloral nectar, or plant sap. Thus, the evidence for a pollination connection is weak. The relevant points are these: 1) When housed in laboratory cages with cuttings of various plant species in bloom, the mosquitoes are later found to contain fructose. 2) They are strongly attracted to certain plant species when they are in bloom. 3) A great variety of plant species have been identified as sources of sugar, indicating plant-host promiscuity. 4) There are no direct field observations of this mosquito feeding on the flowers of any putative host plant in the field or in cages. 5) Plant-host-specific DNA in the mosquitoes cannot exclude sources other than flowers of the specified plant. 6) Based on attractiveness or fructose-positivity data, plants most likely to receive biologically relevant mosquito pollination are *A. macrostachya and P. juliflora*. However, the literature indicates that these and other putative host plants have a great many alternative visitors better suited to pollination, so the contribution of *Ae. aegypti* is likely to be quite small or negligible. At this time, it would be best to accept the null hypothesis that *Ae. aegypti* is not a biologically relevant pollinator.

*2) Anopheles gambiae*. This section expands on the *An. gambiae* s.l. (sensu lato) group of human malaria and filariasis vectors in Africa. Increasing knowledge of the systematics of this group has led to the recognition and identification of several distinct species, including *An. gambiae* s.s. (sensu stricto), *An. arabiensis*, *An. coluzzii*, and others. These species often were not recognized in the earlier literature on plant feeding, because they are difficult to identify without genetic methods. Early authors had discounted the possibility that *An. gambiae* females feed on plant sugar in nature (e.g., Muirhead-Thomson 1951), or it does so only rarely, but recent studies have confirmed otherwise. Despite incomplete information on its flower visits, that subject will be reviewed here because of its extreme importance as a disease vector.

Manda et al. (2007a,b) implicated 13 plant species as hosts of *An. gambiae* in western Kenya, based on several measures of mosquitoes housed with cuttings of plant species in one large outdoor cage or separately in smaller cages. When ranked according to mosquito fructose positivity, *P. hysterophorus* (Santa-Maria feverfew)*, Tecoma stans* (yellow trumpetbush), *Senna didymobotrya* (African senna or popcorn senna), *and R. communis* (castor bean) scored highest. The last three plants also provided the highest survival values, all apparently due to extrafloral nectar. With some exceptions, attractiveness, survival value, and accessible sugar are indications that these plants serve as natural sugar sources, but not necessarily from their flowers. Laboratory and mesocosm experiments substantiated the importance of extrafloral nectaries (Gary and Foster 2004; Stone et al., 2012; Nikbakhtzadeh et al. 2016; Ebrahimi et al. 2017). Most notable were associations with *P. hysterophorus* and *S. didymobotrya*, possibly due to olfactory attraction. In the field, parts of these two plant species and also *Lantana camara* were observed being probed by *An. gambiae* and other insects (W.A. Foster unpubl.). Only the flowers of *P. hysterophorus* were seen being probed, yet in some studies this plant yielded little or no nectar (WA Foster unpubl., Ojija et al. 2019). They appear to ignore the flowers of *S. didymobotrya* and probe only its EFNs, plant-hopper honeydew, and guttation fluids on its leaves. *An. gambiae* readily inserts its proboscis into the corollas of the florets of *L. camara* but appears to obtain nectar mainly or only from the green calyces beneath its inflorescences (Nikbakhtzadeh et al. 2016). The attractiveness of *P. hysterophorus* was confirmed by Nyasembe et al. (2012, 2015) in the laboratory, as was that of *R. communis* to a lesser extent. The sugar derived from *R. communis* probably comes almost entirely from prominent extrafloral nectaries on its leaf petioles (Gary and Foster 2004), not its flowers or plant tissues. Pollination of the asymmetric flowers of some *Senna* spp. are presumed to be achieved mainly by the so-called “buzz pollination” of large bees (Marazzi and Endress 2008). In neither field nor laboratory has *An. gambiae* been observed extracting nectar from the flowers of any *Senna* spp., but these plant species do offer it from extrafloral nectaries and planthopper honeydew (W.A. Foster unpubl.).

Using DNA bar-coding to determine plant associations of *An. gambiae* s.l., Nyasembe et al. (2018) surveyed mosquitoes in three Kenya locations. They identified DNA from *R. communis*, *Senna alata*, *S. tora, Leonotis nepetifolia* (lion ear)*,* and *P. hysterophorus.* So far, the first three putative host plants are known to provide sugar to mosquitoes only from extrafloral nectaries (Gary and Foster 2004) or possibly honeydew from plant-hopper infestations (W.A. Foster unpubl.), not from flowers. *R. communis* generally is self-pollinating or wind-pollinated, though many insects including wasps, bees, flies, and even ants sometimes visit its flowers. Honey bees are considered to be important pollinators in Brazil (Rizzardo et al. 2012), and house flies contribute substantially to its cross-pollination in Cameroon (Douka and Fohouo 2014). As noted previously, *Senna* spp. flowers generally are structured for nectar feeding and pollination by bees, though the flowers of some species of *Senna* possibly are fed upon by mosquitoes as well. So far, that has not been seen, despite numerous observations (W.A. Foster unpubl. data). The fourth DNA-identified host plant, *L. nepetifolia*, has deep corollas with nectar inaccessible to mosquitoes. In its native Africa it is pollinated by sunbirds and in the Americas by hummingbirds and sometimes bees (Raju 2005). Mosquitoes can obtain sugar from the calyces of dehisced senescent flowers (W.A. Foster unpubl.), a stage unlikely to be connected to pollination. By contrast, the fifth DNA-identified host plant, *P. hysterophorus*, has small white composite flowers typical of generalist-pollinated plants. Its odor is attractive to *An. gambiae* s.s. (Nyasembe et al. 2012; Nikbakhtzadeh et al. 2014), and mosquitoes often bear pollen grains on their bodies after probing *P. hysterophorus* flowers (W.A. Foster unpubl.).

Gouagna et al. (2010) proposed the following species as plant hosts of *An. gambiae* in Burkina Faso, based on their proximity, fructose contents, and olfactory attraction: *Mangifera indica* (mango), *Delonix regia* (flame tree)*, Thevetia neriifolia* (= *T. peruivana)* (= *Cascabela thevetia*)] (yellow oleander), *Senna siamea*, and *Cassia sieberiana*. Their flowers are reported to be visited and pollinated by a wide variety of insects, including bees, wasps, butterflies, and large flies, but also by birds (e.g., Fajardo et al. 2008; Suraliwala and Gopalkrishnan 2021; Lopez-Uribe et al. 2008). Others have extrafloral nectaries, e.g., mango trees (Marohomsalic et al. 2021), which are alternative sources of fructose. Feeding on the flowers has been implied but not reported or described. In La Reunion, Gouagna et al. (2014) implicated the following species as plant hosts of *An. arabiensis*: *Duranta erecta*, *Stachytarpheta urticifolia, Leucocaena leucocephala*, *Centratherum punctatum*, *Leucas lavandufolia*, and *Lantana camara*, based on larval site, peridomestic location, laboratory attraction, or acquisition of fructose from their flowers in cages. Actual feeding from the flowers in the laboratory or field was not reported. Most have tubular flowers whose nectar is usually inaccessible to mosquito mouthparts and are known to be visited and pollinated by a wide variety of insects, including bees, wasps, butterflies, and large flies (e.g., Thakur and Mattu 2010; Barbola et al 2006). Such a wide variety of implicated plant hosts, and such a diversity of alternative pollinators, suggests that *An. arabiensis* ‘s contribution to pollination of these plants is trivial or redundant.

In Mali, Müller et al. (2010a, b) tested *Anopheles gambiae* flower attraction to 26 plant species in the field. They found that *A. macrostachya, A. albida, Boscia angustifolia, Ziziphus mauritiana, Guiera senegalensis, A. senegal, A. nilotica, Leptadenia pyrotechnica, and Crotalaria* sp. were attractive. *A. macrostachya* had the highest attraction index for both sexes. This indigenous plant species has threatened status in some areas of West Africa (Gaisberger et al. 2017). Direct field observations overnight on *A. macrostachya* during its flowering season in The Gambia revealed that all feeding on this tree by mosquitoes of several species, including *An. gambiae* and *Ae. aegypti*, occurred on extrafloral nectaries of the acacia’s leaf petioles, not on its closely associated flowers (A.W.R. McCrae pers. comm.). Honey bees are reported to be among its pollinators. Flowers of *Acacia* spp. in general are visited by a wide variety of insects that probably serve as its pollinators (see Montesinos et al. 2016).

Also in Mali, Müller et al. (2017) removed the flowering branches of the invasive species *Prosopis juliflora*, resulting in a 69% drop in *Anopheles gambiae* abundance. This is evidence of a strong flower constancy and nectar dependence at the time of the experiment, probably a result of its high attractiveness and the paucity of competing sources of nectar in an arid region of Mali during the dry season. The major flower visitors of this tree are long-tongued bees and syrphid flies, but also include a wide variety of beetles, wasps, flies, moths, and butterflies (Sajjad et al. 2012; Hussain et al. 2021). Thus, *An. gambiae* may be heavily dependent on this plant when sugar is scarce, but this relationship is one-way. *P. juliaflora* probably makes use of many different pollinators, so the vector’s role in pollination is likely to be minor, trivial, or redundant.

In Bukina Faso, the ability of *An. coluzzii* to obtain fructose from plant baits within cages was used to examine suspected plant hosts: *Mangifera indica* (mango) fruit, *Lannea microcarpa* (African grape) fruit, *T. neriifolia* flower cuttings *(*= *T. peruivana)* (= *Cascabela thevetia*), and flowering cuttings of *Barleria lupilina* (hop-headed barleria) (Hien et al. 2016). No direct observations of nectar feeding or fruit piercing were reported, so the method and source of sugar remains unclear. Native bees and butterflies are considered to be among *T. neriifolia*’s and *B. lupilina’s* important pollinators (e.g., Lopez-Uribe et al. 2008).

In outdoor enclosures in western Kenya, Yalla et al. (2023) measured the attractiveness of a variety of local flowering plants to *An. gambiae* and two other Anophelines, using glue-netted traps baited with bunches of flower cuttings of each species. *Mangifera indica* (mango, an economically important fruit-bearing tree)*, Crotolaria pallida, Tithonia diversifolia* (Mexican sunflower)*, Makhamia lutea, and P. hysterophorus* (Santa-Maria feverfew) were all attractive. The odor of mango flowers were ranked as most attractive. The behavior of flower visits was not reported, so flower-nectar feeding is not certain. Extrafloral nectaries of these and other potential host plants were not considered, nor were other insects that visit the flowers. The literature records a great variety of mango pollinators in the insect orders Hymenoptera, Diptera, Lepidoptera, and Coleoptera, but mosquitoes were not listed among them (Ramirez and Davenport 2016). A singular beneficial feature of the attractive invasive pest plant *P. hysterophorus* is its prodigious supplies of pollen for honey bees in India. It is considered a valuable food resource for honey bee hives when other sources of pollen are scarce (Bhusari et al. 2005; Dalio 2013). In addition to mosquitoes, a very wide variety of other insects visits its flowers and are presumptive pollinators, including flies, wild bees, wasps, beetles, butterflies, and hemipterans. Its pollen also is thought to be dispersed by wind (Usharani and Solomon Raju 2018; Ojija et al. 2019). Therefore, although anophelines and culicines are reasonable candidates for biologically relevant mosquito pollination, there is no persuasive evidence of this, and their contribution is likely to be trivial, redundant, or nil.

*Summary: An. gambiae* s.s. and s.l. are confirmed feeders on plant sugar in the field, as revealed by their fructose contents. Whether much of it comes from flowers, however, is unknown. Honeydew, extrafloral nectar, and plant sap, which are not connected to pollination, are common alternative sugar sources. As with *Ae. aegypti*, the evidence for a flower association comes mainly from weak and indirect observations. The salient points are these: 1) When housed in laboratory cages with cuttings of various plant species in bloom, the mosquitoes are later found to contain fructose. 2) They are strongly attracted to certain plant species when in bloom. 3) Direct observations of *An. gambiae* while probing flowers have been seen only on the invasive plant *P. hysterophorus*. 4) The identity of plant-specific DNA in the mosquitoes cannot exclude sources other than flower nectar or tissues from the specified plant, due to cross-contamination. 5) Many plant species have been suggested as possible plant hosts of *An. gambiae*, implying plant-visit promiscuity. 6) Nearly all instances of direct observation of probing and feeding in cages and in the field have been on extrafloral nectaries or honeydew, not on flowers. Among the plant species whose flowers seem likely to be visited by *An. gambiae—e.g., A. macrostachya, P. juliflora, M. indica,* and *P. hysterophorus—*their presumed or confirmed pollinators include a great variety of other insects. The literature on these plants and their flower visitors lacks any mention of mosquitoes, indicating that *An. gambiae* and other mosquitoes are greatly outnumbered, both in species and in individuals, by visitors better suited for pollination. One may conclude that the contribution of *An. gambiae* to their pollination, if any, is likely to be minor, trivial, or redundant. At this time, it would be best to accept the null hypothesis that* An. gambiae* is not an important pollinator.

## Discussion

### A. The invasive-weed and invasive-vector coincidence

An irony of *Ae. aegypti* and *An. gambiae* relationships with plants is that many appearing most likely to be pollinated by these vectors also are destructive, invasive, pest plants (Stone et al. 2018 review). *P. hysterophorus* and *P. juliaflora*, two spreading alien plant species, are notorious examples. Their flowers are very attractive to both *An. gambiae* and *Ae. aegypti*. *P. hysterophorus* chokes agricultural fields, displaces native plant communities, depletes pollen flow of native plants by interfering with their pollinators (Usharani and Solomon-Raju 2018; Ojija et al. 2019), is toxic to livestock (Kumar et al. 2009; Dalio 2013), and has been proposed to promote malaria transmission (Nyasembe et al. 2015). Furthermore, it is reported to be pollinated by a wide variety of insects, including bees, ants, flies, butterflies, and beetles (Ojija et al. 2019). Field studies indicate that *P. juliaflora* enhances *An. gambiae* populations (see Müller et al. 2017), displaces native plants, is toxic to livestock, and makes land unusable (William and Jafri 2015; Sintayehu et al. 2020). Like *P. juliaflora, P. hysterophorus* is reported to be pollinated by many kinds of insects (Sajjad et al. 2012). Thus, these top disease vectors are unlikely to be more than trivial or redundant pollinators of these weeds unless their densities are disproportionately high.

Among the invasive vectors, *Ae. aegypti* is an example of an eminently successful one, with both disease-transmitting ability and possible pollination potential. It has become established in nearly all human-disturbed tropical, subtropical, and mild temperate climates throughout the world, often propagating serious pathogenic viruses in these places (Lounibos 2002). Its main natural sources of plant sugar await confirmation. The studies of Nyasembe et al. (2018) and Sissoko et al. (2019) suggest that *Ae. aegypti* shares many of the same plant species with *An. gambiae*, which also concentrates near human habitations. *Ae. aegypti* has an even stronger proclivity for the peridomestic environment, which provides development and resting sites in the immediate vicinity of its main blood supplies and mating foci, that is, humans. Thus, invasive plants and invasive disease vectors appear to go together. Nonetheless, we have no convincing evidence that a weed-vector mutualism exists.

### B. The mosquito-control issue

If mosquitoes were not a major source of irritation and disease, details of their lives would receive little attention. Instead, knowledge of their place in the scheme of community ecology is central to sensible mosquito control, and their possible role as pollinators of plants is a topic worth examining. Yet, despite an increasing awareness of the need to minimize the impact of insecticides and habitat alteration on honey bees, wild pollinators, predators, parasitoids and other beneficial insects (see Ginsberg et al 2017; Khallaayoune et al. 2013), the possible pollinating abilities of mosquitoes are rarely addressed. Perhaps the topic will receive more study, as novel genetic methods of control become acceptable, by offering less destructive ways to selectively eliminate a mosquito species from regions bearing a heavy burden of mosquito-borne disease.

In a few mosquito-plant relationships, such as the orchid *P. obtusata,* mosquitoes are *confirmed* and *important* to a plant’s pollination, meeting the criteria of Adams and Lawson (1993) and sustaining the plant’s reproductive success. A few more may be implicated as cases of *possible* or *probable* pollination, owing to their frequent visits to flowers. However, the great majority of mosquito-plant relationships are even less certain, failing to meet even the criteria of a *possible* pollinator in the sense of Adams and Lawson (1993)—without even considering their biological relevance. Many of those relationships include information that argues against a biologically important pollination role.

The argument pertains especially to the promiscuity of either the plant or the mosquito, usually both. Mosquito vectors of disease, like nearly all mosquito species, frequent generalist flowers, and they are almost always polylectic. The flowers they visit are visited by many other insects with better pollination potential. In addition, their common use of non-floral sugar sources in the tropics and subtropics mitigates opportunities for pollen transfer. That likely puts *An. gambiae* and *Ae. aegypti* contributions to pollination of most plants in one of the least effective categories: minor, trivial, redundant, or nil. Thus, the pollination hypothesis as it applies to top vectors has very little support and should be dismissed until intensive investigations provide new and favorable evidence to the contrary.

The knowledge outlined in the sections above make a persuasive case that very few mosquito species of any kind contribute even marginally to pollination success. Thus, concern about the impact of vector control or elimination on pollination might be thought unworthy of consideration. However, the subject of vector pollination should not be ignored completely. It may be argued that even a minor or trivial reduction in pollination caused by the absence of one mosquito species might make a small difference to the reproductive success of a plant species, with potentially cascading or resonating ecological effects. For example, even if its contribution to maximum seed set were only 2%, we could not confidently predict that its effect on a plant’s reproductive success would be redundant or nil without a full knowledge of the pollination networks of the entire insect-plant community (e.g., Olesen et al. 2007; Astegiano et al. 2015), a major undertaking. The outcome of lowering average seed set by only 2%, due to the absence of one pollinator, is probably inconsequential compared to stochastic variations in plant reproduction from year to year for other reasons. So while the effect is plausible, a complete knowledge of all mosquito-plant associations and their roles in a complex pollination system would be necessary to allow firm conclusions. A synthesis of the current knowledge of mosquito-plant associations, potential pollinators, and historical precedent strongly suggests that the suppression or disappearance of a single mosquito species from a location will not affect generalist plant species, and by itself will not perturb the composition of their biotic communities.

If either vector were found to be a valuable pollinator of agricultural crops, native plants, or useful landscape plants, the costs and consequences of focal suppression or elimination of that vector, and thus reduced reproductive success of its favored plants in a particular region would have to be weighed against the improved human health and life in the same region. In making that assessment the following perspectives are useful: (1) The vector favors invasive pest plants as sources of sugar. If it pollinates these weeds, it helps to maintain or spread them, thus ruining agricultural land and altering natural biotic communities. (2) The vector invades and thrives in human settlements with disturbed landscapes. It is not a natural part of stable communities of native plants. Its effectiveness as a disease vector is favored by human activities that create man-made habitats and alters vegetation that may provide new sources of sugar. (3) The vector spreads and maintains human diseases such as malaria, filariasis, yellow fever, dengue, Zika, and Chikungunya, all of them causing sickness, suffering, malformation, or death. These infections place a heavy economic burden on human populations. (4) The vector offers an ideal target for narrowly-focused methods of vector control. Its association with human settlements and housing facilitate suppression or elimination, because its densities can be more easily reduced to a point where pathogen transmission is unsustainable. (5) The reduced use of chemical insecticides and land alterations to suppress vectors temporarily, when methods such as genetic control are implemented, likely offsets conceivable harm caused by pollination-related changes in the composition or structure of biotic communities.

Our ability to foresee all the consequences of suppressing or eliminating a vector must depend on a much greater knowledge of a vector’s pollination prospects than we currently have, without even considering its whole pollination network in its community and its ecosystem. Giving pollinator status to a vector that visits some flowers without more information than currently available is inadvisable. Nevertheless, we may speculate that, given what little we know at the moment about the plant preferences of *An. gambiae* and *Ae. aegypti* and their potential pollinating abilities, a narrowly targeted suppression or elimination of their populations conceivably could have three outcomes: (1) reduced density of some plant populations or alterations in their communities, (2) reduced density of damaging invasive-weed populations, or (3) interruption of the transmission of serious human diseases.

## Conclusion

Current evidence supports the conclusion that the snow-melt mosquito *Aedes communis* is an *effective* and important pollinator of the orchid *P. obtusata* but not the only one. A few other mosquito species probably are contributing pollinators in some locations, and their roles may be accepted provisionally, pending further investigation and application of exacting criteria. Nonetheless, the vast majority of the ~ 3,500 mosquito species on earth appear to contribute little or almost nothing to the reproductive success of the plants they visit. The hypothesis that they are consequential pollinators must be rejected without strong evidence indicating otherwise. This conclusion applies particularly to the notorious disease vectors *An. gambiae* and *Ae. aegypti*. The benefits of suppressing or eliminating either of them from specified regions appear to outweigh conceivable negative ecological consequences that might result from the reduction of their speculated pollinating services.
